# ATM meets ERK5

**DOI:** 10.18632/aging.101189

**Published:** 2017-02-27

**Authors:** Maria Angulo-Ibáñez, Xavier Rovira-Clavé, Enric Espel

**Affiliations:** Celltec-UB, Department of Cell Biology, Physiology and Immunology, Faculty of Biology, University of Barcelona, Barcelona, Spain

**Keywords:** cell cycle, T-cell development, cancer

The complexity of the living cell is supported by the multitasking activity of its protein constituents. The kinase Ataxia Telangiectasia Mutated (ATM) is a clear example. Not only does it orchestrate the DNA damage response but also sustains cellular homeostasis, including metabolism, control of oxidative stress, autophagy and apoptosis [1]. Given its prevalent role in the cellular response to various forms of stress, it is not surprising that mutations that inactivate ATM occur in ∼10% of human cancers. The mitogen-activated protein kinase (MAPK) ERK5 also presents an obvious multitasking activity. Besides being a nodal point to growth factor and stress signaling pathways, ERK5 has a C-terminus with transcriptional coactivator activity [2]. ERK5 mutations in cancer are a rare event, however, an increasing body of evidence relates ERK5 to cancer. ATM can phosphorylate a vast network of cellular substrates, but ERK5 is not in the list of close relatives. Nevertheless, the two kinases might be closer than expected. *Atm*^−/−^ mice, like humans carrying *ATM* inactivating mutations, are prone to lymphomagenic tumors. Recently, we have demonstrated that in absence of ERK5 the development of T-cell lymphoma in *Atm*^−/−^ mice is reduced, revealing a functional link between ATM and ERK5 [3].

**Figure 1:**
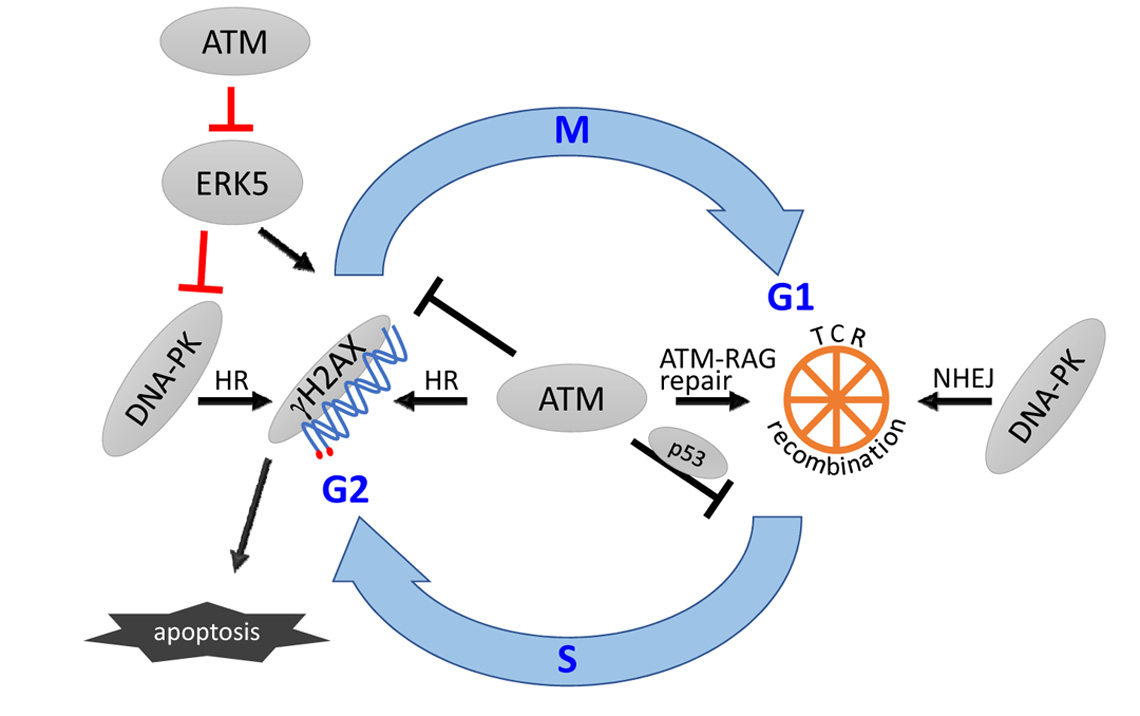
A schematic model depicting the hypothetical role of ERK5 (red bars) in the cellular response to unrepaired DNA damage in VDJ recombination in thymocytes. NHEJ, non-homologous end-joining pathway of DSB repair. HR, homologous recombination. gH2AX, phosphorylated H2AX. ATM-RAG, indicates the cooperation between both proteins to stabilize the DSB. The bars indicate inhibitory function.

The loss of ERK5 presents minor effects in T cell development in mice [4], whereas the loss of ATM profoundly affects it, thanks to ATM's involvement in the T-cell receptor (TCR) recombination process and repair of unresolved DNA double strand breaks (DSB). The recombination of VDJ gene segments of *TCR* takes place in the G1 cell cycle phase of the most immature T cells, namely CD4^-^CD8^-^ double-negative (DN) thymocytes, initiated by the lymphocyte-specific endonuclease RAG1/2. To prevent cell division in thymocytes with unrepaired DSB, ATM activates G1/S and G2/M cell cycle checkpoints. However, in absence of ATM, the G1/S and G2/M checkpoints are less effective, some VDJ DSB escape G1 and evolve into chromosomal translocations. The presence of DSB activates the phosphorylation of histone H2AX by ATM and DNA-dependent protein kinase (DNA-PK). Phosphorylation of H2AX is necessary to preserve the structural integrity of DNA ends, for proper functioning of the G2/M checkpoint and apoptosis in response to genomic stress [5]. Our recent results show that the phosphorylation of H2AX and apoptosis in response to DSB are impaired in *Atm*^−/−^ mice but not in *Atm*^−/−^*Erk5*^−/−^ thymocytes, suggesting that ERK5 inhibits H2AX phosphorylation in *Atm*^−/−^ mice [3]. Moreover, *Atm*^−/−^*Erk5*^−/−^ mice presented a higher percentage of DN thymocytes in the G2/M cell-cycle phase. We therefore envision a model where the phosphorylation of H2AX by DNA-PK in response to unrepaired DSB in thymocytes of *Atm*^−/−^ mice is inhibited by ERK5, thereby lessening the G2/M checkpoint and increasing genomic instability (Figure 1). In wild type mice, this ERK5 activity would be under control of ATM in a signaling pathway regulating DNA repair, G2/M checkpoint and apoptosis.

The delayed death due to T-cell lymphoma development in *Atm*^−/−^*Erk5*^−/−^ mice suggests that pharmacological targeting of ERK5 has therapeutic potential for T-cell lymphoma patients carrying inactivating *ATM* mutations. However, as ERK5 participates in additional cellular functions, the strategy that involves the use of ERK5 inhibitors needs to be carefully evaluated. For instance, we have recently shown that ERK5 modulates the dCTP levels in T cell acute lymphoblastic leukemia Jurkat cells treated with thymidine, thereby reducing DNA replication stress [6]. Therefore, if ERK5 inhibitors are to be used in combination with antineoplastic dCTP analogues such as cytarabine, the former could counteract the therapeutic effect of the latter [7]. The variety of cellular functions governed by ERK5 and ATM constitute a barrier for therapy, warranting a search for specific substrates of these kinases as potential targets for therapy.
